# Mechanism of sodium butyrate, a metabolite of gut microbiota, regulating cardiac fibroblast transdifferentiation via the NLRP3/Caspase-1 pyroptosis pathway

**DOI:** 10.1186/s13019-024-02692-0

**Published:** 2024-04-15

**Authors:** Tiancheng Dong, Dingkao Huang, Zhengzheng Jin

**Affiliations:** Department of Intensive care unit, Wenzhou TCM Hospital of Zhejiang Chinese Medical University, No. 9 Liuhongqiao Jiaowei Road, Wenzhou City, 325000 Zhejiang Province China

**Keywords:** Gut microbiota metabolite sodium butyrate, Cardiac fibroblasts, Myofibroblasts, NLRP3 inflammasomes, Caspase-1, Cell pyroptosis, TGFβ1, α-SMA

## Abstract

**Background:**

Cardiac fibroblasts (CFs) are activated after initial injury, and then differentiate into myofibroblasts (MFs), which play a pivotal role as the primary mediator cells in pathological remodeling. Sodium butyrate (NaB), being a metabolite of gut microbiota, exhibits anti-inflammatory property in local therapies on sites other than the intestine. Thus, this study aimed to probe the mechanism by which NaB regulates CFs transdifferentiation through the NLRP3/Caspase-1 pyroptosis pathway.

**Methods:**

CFs were cultured in vitro and induced into MFs by TGFβ1. CFs were identified by immunofluorescence labelling technique of vimentin and α-SMA, followed by treatment with NaB or NLRP3 inflammasome inhibitor (CY-09) and its activator [nigericin sodium salt (NSS)]. The expression levels of α-SMA, GSDMD-N/NLRP3/cleaved Caspase-1 proteins, and inflammatory factors IL-1β/IL-18/IL-6/IL-10 were determined using immunofluorescence, Western blot and ELISA. Cell proliferation and migration were evaluated using the CCK-8 assay and the cell scratch test, respectively.

**Results:**

Following the induction of TGFβ1, CFs exhibited increased expression levels of α-SMA proteins and IL-6/IL-10, as well as cell proliferative and migratory abilities. TGFβ1 induced CFs to differentiate into MFs, while NaB inhibited this differentiation. NaB inactivated the NLRP3/Caspase-1 pyroptosis pathway. CY-09 demonstrated inhibitory effects on the NLRP3/Caspase-1 pyroptosis pathway, leading to a reduction in TGFβ1-induced CFs transdifferentiation. NSS activated the NLRP3/Caspase-1 pyroptosis pathway, and thus partially counteracting the inhibitory effect of intestinal microbiota metabolite NaB on CFs transdifferentiation.

**Conclusion:**

NaB, a metabolite of the gut microbiota, inhibited the activation of the NLRP3/Caspase-1 pyroptosis pathway in TGFβ1-induced CFs, repressed the transdifferentiation of CFs into MFs.

## Background

The harboring trillions of microorganisms that reside in the intestines is referred to as the gut microbiota [1]. With the ongoing advancement and refinement of metabolomics and microbial research technology, there has been a growing body of research on the impact of gut microbiota and its metabolites on body health, among which short chain fatty acid (SCFAs) are metabolites of gut microbiota that are small molecule compounds produced by microbial saccharification and fermentation of dietary fiber, mainly including acetic acid, propionic acid, butyric acid, isobutyric acid, valeric acid, isovaleric acid and hexanoic acid [2]. Notably, SCFAs have shown the capacity to preserve the stability of the intestinal mucosal barrier, promote colon cell proliferation, and trigger terminal differentiation and apoptosis of epithelial cells [3, 4].

Microbiota-derived metabolites, known as SCFAs, including acetate, butyrate, and propionate, are primarily generated through bacterial fermentation of dietary fiber within the gastrointestinal tract [5]. A prior study has demonstrated that high dietary fiber diet can improve the physiological parameters of mice with myocardial hypertrophy and alleviate cardiac fibrosis [6]. SCFAs exhibit a potent anti-inflammatory effect and play a role in the pathogenesis of cardiovascular disease [7]. Sodium butyrate (NaB), an important member of SCFAs, is synthesized through the bacterial fermentation of non-digestible carbohydrates [8]. NaB can be delivered from the gut to organs such as the heart, liver, and lungs, and increased intestinal NaB production leads to an augmentation in NaB circulation level [9]. In addition, NaB maintains the balance of the gut-heart axis [10]. Meanwhile, NaB also plays a pivotal part in the pathogenesis of cardiovascular disease [5, 11].

Cardiac fibroblasts (CFs) exist in a static form in the healthy heart, but differentiate into myofibroblasts (MFs) in response to various stimuli such as inflammation and trauma, and are typically characterized by upregulation of α-smooth muscle actin (α-SMA) expression, and elevated cell migration and proliferation [12, 13]. Transforming growth factor β1 (TGFβ1) is a well-recognized growth factor that drives the differentiation of CFs into MFs, leading to a boosted α-SMA protein expression in the cells, and the secretion of biologically active molecules such as interleukin (IL)-6, IL-10, and other inflammatory cytokines [14, 15]. It has been documented that repression of the Caspase-1/Gasdermin D (GSDMD) axis reduces myocardial pyroptosis and inhibits transdifferentiation of CFs [16]. Suppression of nucleotide-binding oligomerization domain-like receptor with pyrin domain 3 (NLRP3) expression and Caspase-1-activated nucleotide-binding oligomerization domain-like receptor in cardiac tissue subdued the transformation of CFs into MFs [17].

NLRP3 inflammasome is a multimeric cytoplasmic protein complex composed of NLRP3, apoptosis-associated speck-like protein ASC and caspase-1 [18]. The activation of NLRP3 inflammasome prompts ASC to recruit pro-caspase-1, leading to the auto-catalytic activation of caspase-1, and the cleaved caspase-1 cleaves GSDMD into the N-terminal domain of GSDMD (GSDMD-N) and forms pores on the plasma membrane to facilitate the maturation and release of IL-1β and IL-18, thereby causing pyroptosis [18, 19]. Proir studies have demonstrated that supression of the NLRP3/Caspase-1 pathway can not only significantly inhibit the formation of fibrosis after acute kidney injury, but also induce hepatocyte pyroptosis to curb hepatocyte fibrosis [20, 21], indicating that the NLRP3/caspase-1 axis-mediated pyroptosis is closely bound up with the process of cell fibrosis. Moreover, NaB impedes high glucose-induced NLRP3 overexpression in human monocyte macrophage THP-1 cells, negatively regulates NLRP3-mediated inflammatory signaling pathways, suppresses macrophage activation and secretion of pro-inflammatory mediators (such as IL-18 and IL-1β), reduces intestinal inflammation level, and limits colitis-associated cancer development [22, 23]. Both the NaB signal and the GPR43 signal after binding to NaB activate NLRP3 inflammasome, and thus producing more IL-18 [24], suggesting that NaB exhibits a close association with the NLRP3 inflammasome and its mediated inflammatory pathway. However, there is a dearth of reports in regard to whether NaB can regulate CFs transdifferentiation through the NLRP3/Caspase-1 pyroptosis pathway, and its mechanism. This study seeks to examine the underlying mechanism by which NaB, a metabolite of gut microbiota, regulates CFs transdifferentiation via the NLRP3/Caspase-1 pyroptosis pathway.

## Methods

### Culture and grouping of CFs

Mouse CFs (FH-M187) obtained from Fuheng Biology (Shanghai, China) were maintained in mouse CFs complete culture medium (PYM188, Fuheng Biology) at 37 °C, with 95% humidity and 5% CO_2_. After 3 days of cultivation, the morphology of primary CFs was observed under a microscope (Olympus CKX41, Olympus, Tokyo, Japan). CFs transdifferentiation was induced by treating the cells with TGFβ1 (10 ng/mL; P01137, MedChemExpress, Monmouth Junction, NJ, USA) for 24 h [14, 25, 26].

The CFs were grouped as follows: the CFs group (normally-cultured CFs), the CFs + fetal bovine serum (FBS) group [CFs cocultured with an equal amount of 10% FBS (solvent control of TGFβ1) for 24 h], the CFs + TGFβ1 group [CFs cocultured with TGFβ1 (10 ng/mL) for 24 h [14]], the CFs + TGFβ1 + NaB group [CFs cultured with 10 ng/mL TGFβ1 and 11 mg/mL NaB [303410-5G, Sigma-Aldrich, St. Louis, MO, USA) for 24 h [27]], the CFs + TGFβ1 + CY-09 group [CFs cultured with TGFβ1 (10 ng/mL) and 1 µM NLRP3 inflammasome inhibitor (CY-09; ab287086, Abcam, Cambridge, UK) for 24 h] [28], the CFs + TGFβ1 + dimethyl sulfoxide (DMSO) group [CFs cultured with TGFβ1 (10 ng/mL) and 1 µM CY-09 solvent (DMSO) for 24 h], the CFs + TGFβ1 + nigericin sodium salt (NSS) group [CFs cultured with TGFβ1 (10 ng/mL) and 5 µM NLRP3 inflammasome activator (NSS; ab120494, Abcam) for 24 h] [29], the CFs + TGFβ1 + NaB + NSS group [CFs cultured with TGFβ1 (10 ng/mL), 11 mg/mL NaB and 5 µM NSS for 24 h], and the CFs + NSS group (CFs cultured with 5 µM NSS for 24 h).

### Immunofluorescence

Adherent cells were washed thrice with phosphate buffered saline (PBS) and fixed at room temperature for half an hour with 4% polymethyltransferase. Subsequently, the cells were subjected to 3 times gentle shaking with PBS for 5 min each, using a shaker at room temperature. Thereafter, cells were added with 0.1% Triton X100 in a dropwise manner for 30 min to break the membrane Following this, cells were introduced with 1% bovine serum albumin (BSA) dropwise for blocking at room temperature for 30 min, followed by 3 times gentle shaking with PBS for 5 min each on a shaker at room temperature. Subsequently, cells were fostered with α-SMA (ab7817, abcam) and anti-vimentin (1:100, ab92547, Abcam) primary antibodies overnight at 4°C, and then incubated with goat anti-rabbit immunoglobulin G (IgG) (1:200, Alexa Fluor 488, Abcam) and goat anti-mouse IgG (1:2000, Alexa Fluor 647; Abcam) secondary antibodies at room temperature on a shaker in the dark for 1 h. Afterward, the cells were subjected to staining using 4’,6-diamidino-2-phenylindole and cultivated at room temperature for 30 min. Susequently, the cells were rinsed with PBS 3 times, and observed and captured under a fluorescence microscope (Olympus).

### Western blot

Cells were collected and treated with lysis solution to extract protein, with the protein concentration measured using the bicinchoninic acid kit (AR1189, Boster Biological Technology, Wuhan, Hubei, China). The protein samples were then supplemented with an appropriate quantity of sample loading buffer and heated in a boiling water bath for 5 min in order to facilitate protein denaturation. The denatured protein sample was loaded onto the sample well and subjected to electrophoresis with sodium dodecyl sulfate-polyacrylamide gel electrophoresis. After separation, the protein was transferred onto polyvinylidene fluoride membranes. Later, the membranes were blocked in 3% BSA solution for 2 h, and then added with primary antibodies cleaved caspase-1 (1:10000, #4199, Cell Signaling Technology), NLRP3 (1:2000, ab263899, Abcam) and GSDMD-N (1:2000, #36425, Cell Signaling Technology) overnight at 4 °C, followed by 1-h incubation with horseradish peroxidase-labelled secondary antibody (1:2000, BA1054, Boster Biological Technology) at room temperature in the dark. Enhanced chemiluminescence working solution (AR1191, Boster Biological Technology) was applied for the purpose of development. Image Pro Plus 6.0 software (Media Cybernetics, Silver Spring, MD, USA) was employed to quantitatively quantify the grayscale of each band in Western blot images, with β-actin (1:5000, ab179467, Abcam) serving as the internal reference. The experiment was conducted in triplicate.

### Enzyme-linked immunosorbent assay (ELISA)

Cell supernatant was collected, and the levels of inflammatory factors IL-18 (EMC011.96; NeoBioscience, Shenzhen, Guangdong, China), IL-1β (EMC001b.96.2; NeoBioscience), IL-6 (ab178013, Abcam) and IL-10 (ab185986, Abcam) were measured using ELISA kits in accordance with the manufacturer’s instruction for each specific experimental steps.

### Cell scratch test

The cell scratch test was performed to evaluate cell migration. Differently-treated cells were seeded onto 6-well plates at a density of 1 × 10^4^ cells per well and cultured until reaching 80-90% confluence. Then, an artificial wound was created by scratching the surface using the tip of a P-200 pipette, and cells were cultured in serum-free Dulbecco’s modified Eagle medium. The healing of cell scratches was observed and documented using a microscope (TS100, Nikon, Tokyo, Japan) at 0 and 24 h, and the migration rate of tumor cells was characterized by calculating the percentage of wound width at 24 h compared to wound width at 0 h [30].

### Cell count kit 8 (CCK-8) assay

Cell viability was assessed using the CCK-8 kit (AR1199, Boster Biological Technology). The transfected cells were distributed at a density of 10^4^/well onto a 96-well culture plate. After 48 h of cell growth, the cells were cultivated with 10 µL of CCK-8 solution at 37 °C with 5% CO_2_ for 2 h. The optical density value of each well was measured using a microplate reader (Tecan, Mannedorf, Switzerland) at a wavelength of 450 nm [31].

### Statistical analysis

GraphPad Prism 8.01 (GraphPad Software, San Diego, CA, USA) and SPSS 21.0 (IBM Corp. Armonk, NY, USA) were utilized for data analysis and plotting. The normal distribution of continuous variables were tested by KS. Data conforming to normal distribution were presented in the form of mean ± standard deviation. One-way analysis of variance (ANOVA) analysis was employed for comparisons among groups, followed by the application of Tukey’s test for post-hoc analysis. A value of *P* < 0.05 was regarded as statistically significant.

## Results

### NaB suppressed TGFβ1-induced transdifferentiation of CFs into MFs

**Fig. 1:**
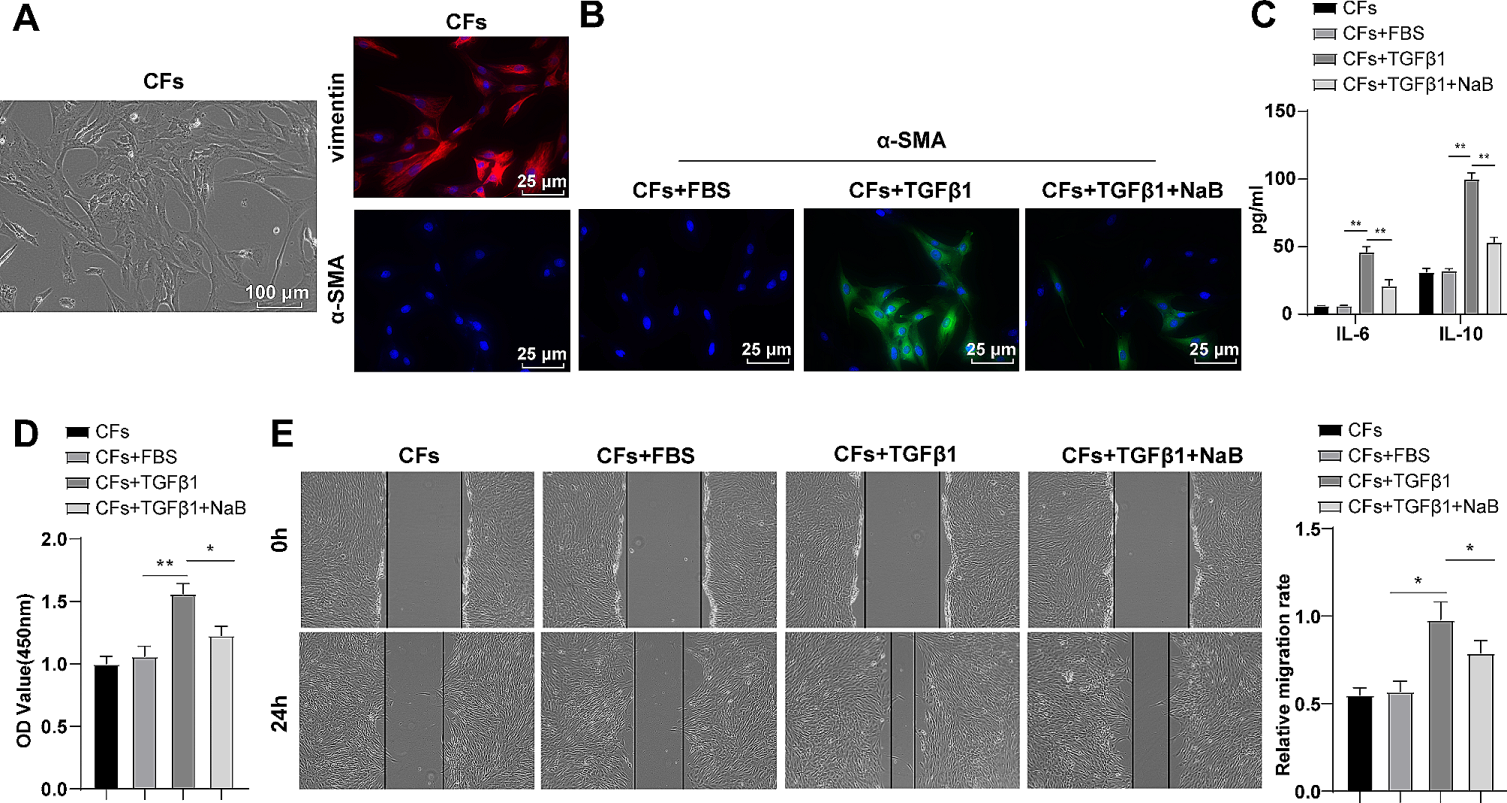
NaB, a metabolite of intestinal microbiota, inhibited TGFβ1-induced proliferation, migration, and transdifferentiation of CFs into MFs. **(A)** Observation of CFs morphology under a microscope, and immunofluorescence labelling of vimentin and α-SMA protein **(B)** Immunofluorescence labelling of α-SMA protein; **(C)** ELISA to measure the expression levels of IL-6 and IL-10; **(D)** CCK-8 assay to evaluate cell proliferation; **(E)** Cell scratch test to assess cell migratory rate. The experiment was repeated three times. The data were expressed as mean ± standard deviation, with one-way ANOVA used for data comparisons among multiple groups, and Tukey’s multiple comparisons test used for post hoc testing. * *P* < 0.05, ** *P* < 0.01

To examine the impact of NaB, a metabolite of gut microbiota, on the transdifferentiation of CFs, CFs were first identified by observation of cell morphology under a light microscopy and by immunofluorescence labelling with vimentin and α-SMA. Most CFs had adhered, with some beginning to extend pseudopodia into spindle-shaped and polygonal shapes; the cell body was large, with weak stereopsis, clear and large nuclei, transparent cytoplasm, in a thin sheet shape, and without spontaneous pulsation; vimentin protein was clearly expressed, whereas α-SMA expression was not expressed (Fig. 1A). Subsequently, CFs were stilumated with TGFβ1 in vitro for 24 h. Immunofluorescence showed that a significant proportion of the cells treated with TGFβ1 exhibited robust expression of α-SMA (Fig. 1B). ELISA determination elicited that the expression patterns of inflammatory factor IL-6 and IL-10 in the CFs + TGFβ1 group were saliently elevated relative to the CFs + FBS group (Fig. 1C, *P* < 0.01). Moreover, CCK-8 and cell scratch test detection manifested enhanced proliferative and migratory abilities of cells in the CFs + TGFβ1 group in contrast to the CFs + FBS group (Fig. 1D-E, *P* < 0.05), indicating that CFs had been differentiated to MFs. Then, CFs were cultured in vitro and treated with TGFβ1, along with NaB for 24 h. Levels of α-SMA protein and inflammatory factors IL-10 and IL-6, as well as cell migratory and proliferative capacity were reduced in the CFs + TGFβ1 + NaB group versus the CFs + TGFβ1 group (Fig. 1B-E, *P* < 0.05). The results suggested that TGFβ1 treatment prompt the differentiation of CFs into MFs, and NaB could limit TGFβ1-induced CFs differentiation into MFs.

### NaB repressed the activation of the NLRP3/Caspase-1 pyroptosis pathway

**Fig. 2:**
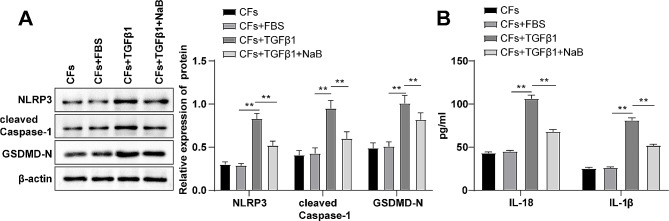
Inhibitory effect of NaB, a metabolite of gut microbiota, on the activation of the NLRP3/Caspase-1 pyroptosis pathway. **(A)** Western blot to test protein expression levels; **(B)** ELISA to assess the expression levels of inflammatory factors. All experiments were repeated three times, with data expressed as mean ± standard deviation. One-way ANOVA was used for data comparisons among multiple groups, followed by Tukey’s test. ** *P* < 0.01

We speculated that as a metabolite of the gut microbiota, NaB might abate the activation of the NLRP3/Caspase-1 pyroptosis pathway. Thus, the expression levels of related proteins in cells were measured by Western blot, which revealed that the levels of NLRP3, cleaved Caspase-1 and GSDMD-N proteins in the CFs + TGFβ1 group were evidently higher than those in the CFs + FBS group, while the levels were apparently lower in the CFs + TGFβ1 + NaB group than the CFs + TGFβ1 group (Fig. 2A, *P* < 0.01). ELISA detection of the expression levels of related inflammatory factors demonstrated that the expression levels of inflammatory factors IL-1β and IL-18 in the CFs + TGFβ1 group were memorably rised compared with the CFs + FBS group, but there was a distinct reduction in the CFs + TGFβ1 + NaB group in contrast to the CFs + TGFβ1 group (Fig. 2B, *P* < 0.01). Overall, NaB could inhibit the activation of the NLRP3/Caspase-1 pyroptosis pathway in TGFβ1-induced CFs in vitro.

### Inhibition of the NLRP3/Caspase-1 pyroptosis pathway reduced TGFβ1-induced CFs transdifferentiation

**Fig. 3:**
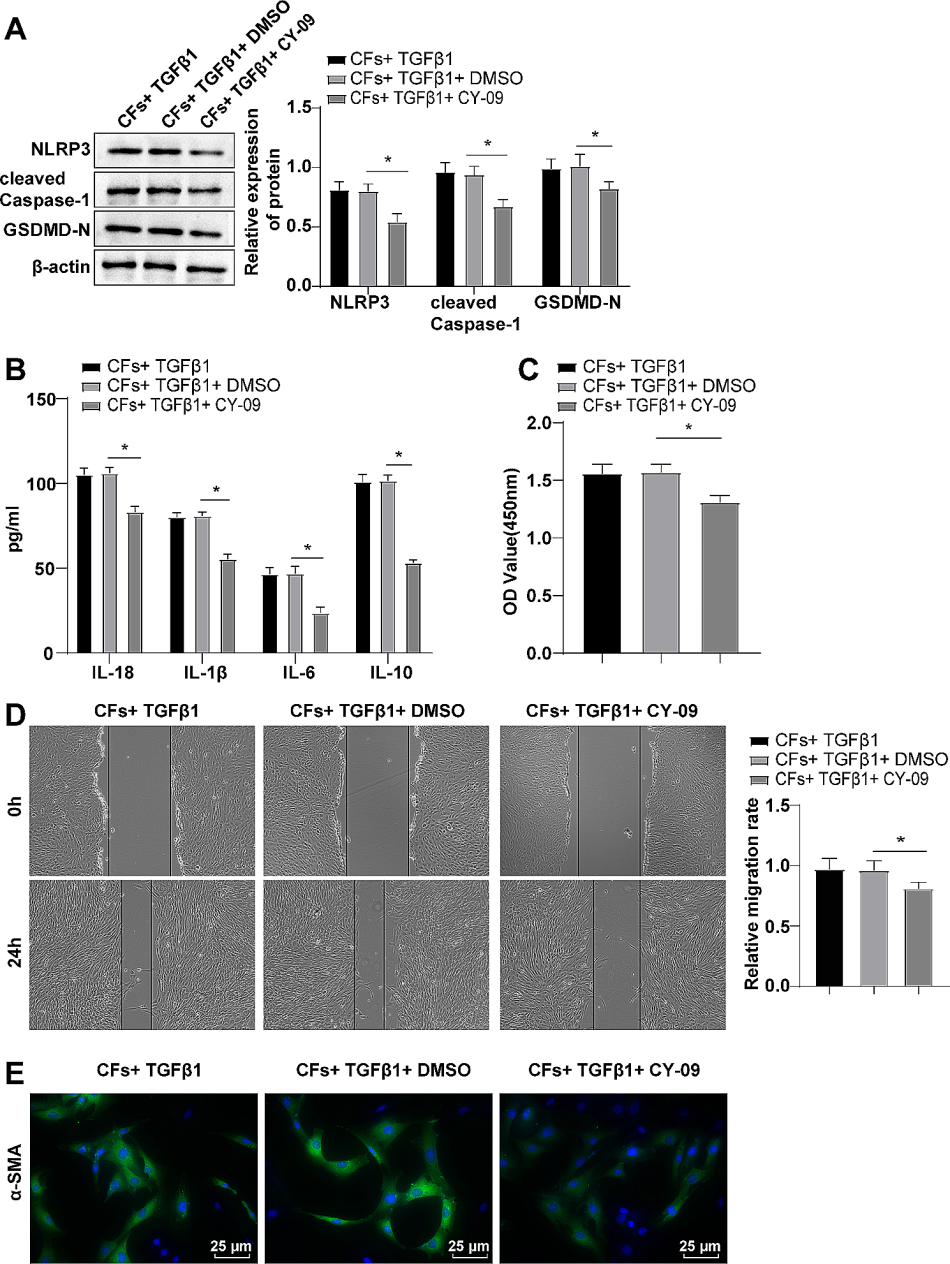
Inhibition of the NLRP3/Caspase-1 pyroptosis pathway reduced TGFβ1-induced CFs transdifferentiation. **(A)** Western blot to measure protein expression levels; **(B)** ELISA to determine the expression levels of inflammatory factors; **(C)** CCK-8 assay to assess cell proliferation; **(D)** Cell scratch test to evaluate cell migratory rate; **(E)** Immunofluorescence labelling of α-SMA protein. The experiment was repeated three times. The data were expressed as mean ± standard deviation, with one-way ANOVA used for data comparisons among multiple groups, and Tukey’s test used for post hoc testing. * *P* < 0.05

Based on the the findings of previous research, we conducted a further investigation to determine if TGFβ1 induces CFs transdifferentiation through the NLRP3/Caspase-1 pyroptosis pathway in activated CFs. Subsequently, cells were subjected to treatment with the NLRP3 inflammasome inhibitor CY-09, while being induced in vitro with TGFβ1. Relative to the CFs + TGFβ1 + DMSO group, the levels of NLRP3, cleaved Caspase-1 and GSDMD-N proteins, as well as inflammatory factors IL-1β and IL-18 were prominently diminished in the CFs + TGFβ1 + CY-09 group (Fig. 3A-B, *P* < 0.05). In addition, upon inhibition of the NLRP3/Caspase-1 pyroptosis pathway, the levels of α-SMA protein and inflammatory factors IL-6 and IL-10, as well as cell proliferation and migration were substantially hindered (Fig. 3A-E, *P* < 0.05). The results indicated that suppression of the NLRP3/Caspase-1 pyroptosis pathway could reduce TGFβ1-induced proliferation, migration and transdifferentiation of CFs.

### Activation of the NLRP3/Caspase-1 pyroptosis pathway partially abrogated the inhibitory effect of the intestinal microbiota metabolite NaB on the transdifferentiation of CFs

**Fig. 4:**
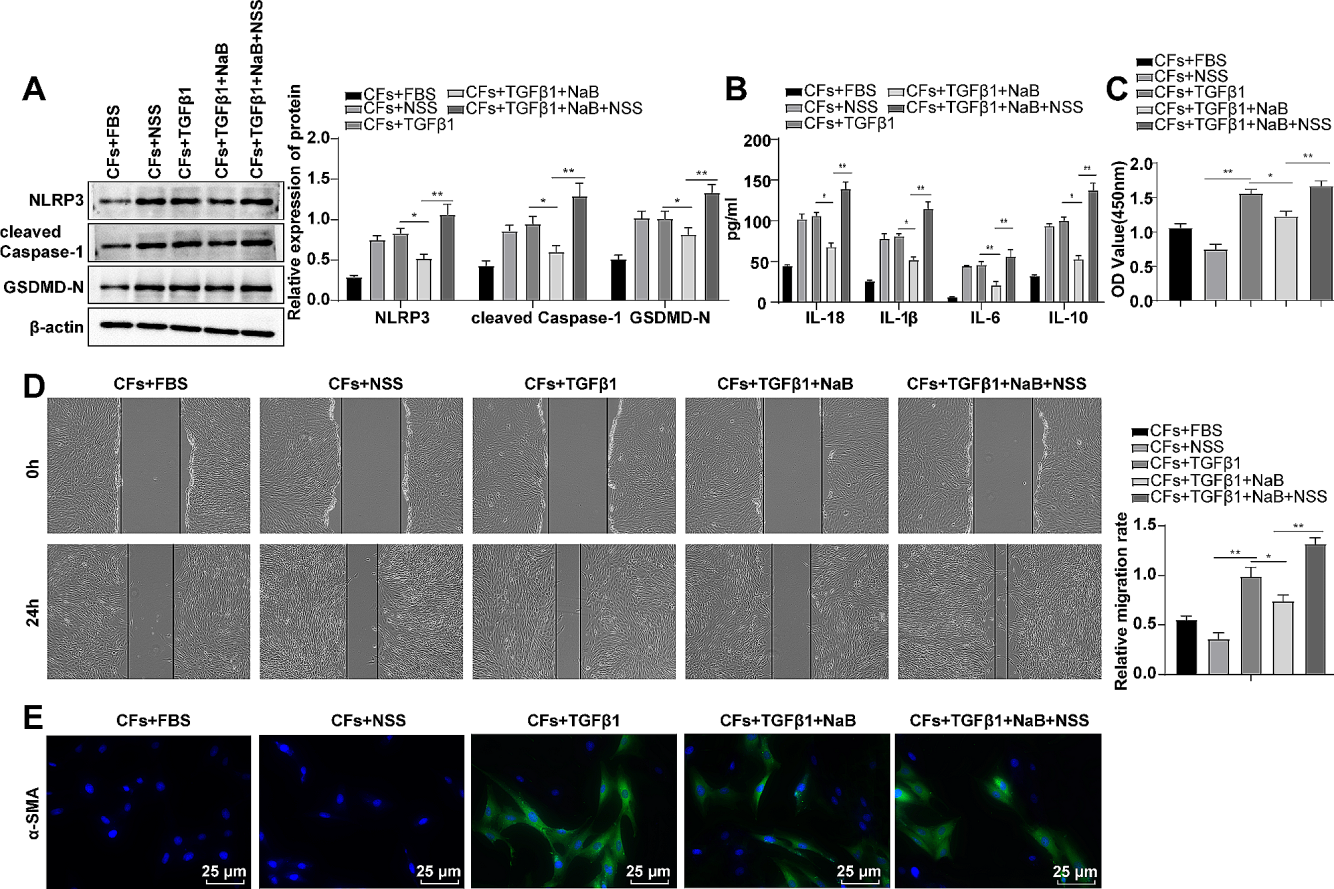
Activation of the NLRP3/Caspase-1 pyroptosis pathway partially nullified the inhibitory effect of NaB, a metabolite of intestinal microbiota, on the transdifferentiation of CFs. **(A)** Western blot to measure protein expression levels; **(B)** ELISA to determine the expression levels of inflammatory factors; **(C)** CCK-8 to assess cell proliferation; **(D)** Cell scratch test to evaluate cell migratory rate; **(E)** Immunofluorescence labelling of α-SMA protein. The experiment was repeated three times. The data were expressed as mean ± standard deviation, with one-way ANOVA used for data comparisons among groups, with Tukey’s test. * *P* < 0.05, ** *P* < 0.01

We treated CFs with NLRP3 inflammasome activator NSS, with the results revealing no prominent differences in NLRP3, cleaved Caspase-1 and GSDMD-N protein levels, and inflammatory factor IL-1β, IL-18, IL-10 and IL-6 levels in the CFs + TGFβ1 group versus the CFs + NSS group (Fig. 4A/B, *P* < 0.05). Besides, proliferative and migratory abilities, and α-SMA protein expression level in cells were distinctly elevated (Fig. 4C-E, *P* < 0.05). The aforementioned results suggested that it was TGFβ1 that had an effect on CFs, rather than the activation of NLRP3 inflammasome itself.

Subsequently, CFs were treated with TGFβ1 and NaB, along with the addition and treatment of the NLRP3 inflammasome activator (NSS). The results unveiled that in contrast to the CFs + TGFβ1 + NaB group, NLRP3, cleaved Caspase-1 and GSDMD-N protein levels, and inflammatory factor IL-1β and IL-18 levels in the CFs + TGFβ1 + NaB + NSS group were measurably up-regulated (Fig. 4A-B, *P* < 0.05), indicating that the NLRP3/Caspase-1 pyroptosis pathway was activated. Meanwhile, after activating the NLRP3/Caspase-1 pyroptosis pathway, and α-SMA protein levels, IL-6 and IL-10 inflammatory factor levels, as well as cell proliferative and migratory capacity were conspicuously intensified (Fig. 4A-E, *P* < 0.05). The aforesaid results indicated that the activation of the NLRP3/Caspase-1 pyroptosis pathway partially annulled the inhibitory effect of NaB, a metabolite of intestinal microbiota, on the transdifferentiation of CFs.

## Discussion

Heart pathological remodeling is a characteristic feature of chronic heart failure and this structural alteration ulteriorly perpetuates the disease, where CFs are the crucial cell type responsible for maintaining heart structural integrity, but stress conditions such as inflammatory and trauma can activate quiescent CFs into contractile and synthetic MFs [13, 32]. SCFAs, produced by the gut bacteria as by-products of the dietary fiber metabolism, have been reported to possess anti-inflammatory properties [33]. As a significant constituent of SCFAs, NaB mitigates renal fibrosis injury and hepatocyte fibrosis [34, 35], suggesting that NaB holds promise as a potential anti-fibrotic agent. But their involvement in CFs transdifferentiation and the underlying mechanism remain largely unexplored. This study highlighted that NaB, as a metabolite of the gut microbiota, limited the activation of the NLRP3/Caspase-1 pyroptosis pathway in CFs induced by TGFβ1 and suppressed the transdifferentiation of CFs into MFs.

The transdifferentiation of CFs into MFs is marked by enhancements in cell migratory and proliferative abilities, as well as an up-regulation of α-SMA expression [12, 13]. TGFβ1 is known to secrete inflammatory cytokines such as IL-10 and IL-6, increases α-SMA protein expression patterns, and to facilitate the transdifferentiation of CFs into MFs [14, 15]. A prior study have indicated that IL-6 exhibits a pro-fibrotic effect [36]. IL-10 has both pro-fibrotic and anti-fibrotic effects, and IL-10 subdues fibrosis in the non-reperfusion myocardial infarction model [37]. In addition, IL-10 displays pro-fibrotic property in myocardial fibrosis response, which may depend on the balance between anti-inflammatory and pro-fibrotic effects [38]. Consistently, we induced CFs transdifferentiation using TGFβ1 and uncovered intensified cell migration and proliferation, as well as elevated levels of IL-10 and IL-6, and α-SMA proteins, while NaB treatment led to contrasting effects in TGFβ1-induced cells. Similar to the results reported by Krishnamurthy P et al. [38], our findings revealed that IL-10 exhibited pro-fibrotic effects in CFs. However, further comprehensive investigations are required to fully understand the intricate mechanisms of IL-10, given its inherent complexity. Likewise, the pre-treatment of NaB obviously represses the expression levels of IL-6 and TNF-α in RAW246.7 macrophages induced by lipopolysaccharide in vitro, suggesting its anti-inflammatory property in inflammatory bowel disease [39]. In addition, post-treatment with NaB dramatically reduces histological changes such as the collagen deposition and fibrosis, while also diminishing the expression levels of α-SMA, iNOS, eNOS, fibronectin, collagen I, NFκB, TGFβ1, DNA damage and apoptosis in the diabetic kidney [40]. Altogether, TGFβ1 treatment induced the transdifferentiation of CFs into MFs, while this trend could be suppressed by NaB.

Exogenous spermidine attenuates diabetic myocardial fibrosis by repressing pyroptosis and inflammation in db/db mice [41]. NLRP3 plays a crucial part in the recognition of exogenous ligands, followed by its downstream effector pro-caspase-1 activation, which cleaves pro-IL-1β, pro-IL-33, and pro-IL-18 to active forms and elicits inflammatory activities [42]. IMD_1 − 53_ provide protection against heart injury and inflammation by attenuating the NLRP3/Caspase-1/IL-1β pathway during sepsis [43]. Our findings disclosed up-regulated GSDMD-N, cleaved Caspase-1 and NLRP3 protein levels, as well as IL-1β and IL-18 levels after TGFβ1 induction, which were then down-regulated by NaB treatment. In line with our findings, it has been reported that the administration of NaB leads to a notable diminishment in relative expression patterns of NLRP3, Caspase-1, IL-18 and IL-1β, suggesting that NaB supplementation have an essential role in diabete alleviation by suppressing pyroptosis [44]. Also, NaB has the potential to ameliorate the condition of renal glomerular endothelial cells induced by high glucose via the Caspase1-GSDMD classical pyroptosis pathway [45]. What’s more, the supplementation of NaB improves the alleviation of lipopolysaccharide-induced diarrhea in mice by decreasing pathogens and enriching beneficial bacterium, which can modulate inflammatory responses and oxidative damages via the NLRP3/Caspase-1 signaling pathway [46]. To conclude, NaB attenuated the activation of the NLRP3/Caspase-1 pyroptosis pathway in TGFβ1-induced CFs. Subsequently, we treated CFs with NLRP3 inflammasome inhibitor CY-09 or NLRP3 inflammasome activator NSS, respectively, and discovered reduced cell migration and proliferation, IL-10 and IL-6 levels, and α-SMA protein levels in response to NLRP3 inflammasome inhibition and TGFβ1 induction, while the results were opposite upon TGFβ1, NaB and NSS treatment. Taken together, our findings innovatively manifested that the inhibition of the NLRP3/Caspase-1 pyroptosis pathway reduced the transdifferentiation of CFs induced by TGFβ1.

However, it is important to acknowledge that there are still certain limitations present in this study. The present study only briefly revealed this mechanism without investigating the role of NaB at the animal and clinical levels. In addition, we exclusively opted for mouse CFs, and did not select more cells for broader research. Consequently, the outcomes of the study in question lack sufficient persuasiveness. miRNAs are closely associated with NLRP3 inflammatory vesicles. As reported, miR-223-3p inhibits fibroblast-like synoviocyte pyroptosis and proliferation and inflammatory responses of fibroblast-like synoviocyte in rheumatoid arthritis by targeting NLRP3 [47, 48]. Inflammasome promotes miR-155 expression, which is a key miRNA driving fibrosis [49]. miR-21 hinders NLRP3 inflammasome activation in lung fibroblasts [50]. In future research, the focus will be on investigating the role of NaB in regulating the upstream miRNAs of NLRP3 inflammasome or the downstream target genes of NLRP3 inflammasome.

## Conclusions

In summary, this study supported that the gut microbiota metabolite NaB attenuated the activation of the NLRP3/Caspase-1 pyroptosis pathway in TGFβ1-induced CFs and reduced the transdifferentiation of CFs into MFs.

## Data Availability

The data that support the findings of this study are available from the corresponding author upon reasonable request.

## Abbreviations

CFs: Cardiac fibroblasts
MFs: Myofibroblasts
NaB: Sodium butyrate
SCFAs: Short chainfatty acid
α-SMA: α-smooth muscle actin
PBS: Phosphate buffered saline
NSS: Nigericin sodium salt
BSA: Bovine serum albumin
IgG: Immunoglobulin G
ELISA: Enzyme-linked immunosorbent assay
CCK-8: Cell count kit 8
ANOVA: Analysis of variance
